# Sex stereotypes influence adults’ perception of babies’ cries

**DOI:** 10.1186/s40359-016-0123-6

**Published:** 2016-04-14

**Authors:** David Reby, Florence Levréro, Erik Gustafsson, Nicolas Mathevon

**Affiliations:** University of Sussex, School of Psychology, Falmer, UK; University of Lyon/Saint-Etienne, Equipe Neuro-Ethologie Sensorielle, ENES/Neuro-PSI CNRS UMR9197, Saint-Etienne, France; Department of Psychology, City University of New York, Hunter College, New York, USA

**Keywords:** Parental behaviour, Sex stereotypes, Vocal communication, Infant cries, Gender

## Abstract

**Background:**

Despite widespread evidence that gender stereotypes influence human parental behavior, their potential effects on adults’ perception of babies’ cries have been overlooked. In particular, whether adult listeners overgeneralize the sex dimorphism that characterizes the voice of adult speakers (men are lower-pitched than women) to their perception of babies’ cries has not been investigated.

**Methods:**

We used playback experiments combining natural and re-synthesised cries of 3 month-old babies to investigate whether the interindividual variation in the fundamental frequency (pitch) of cries affected adult listeners’ identification of the baby’s sex, their perception the baby’s femininity and masculinity, and whether these biases interacted with their perception of the level of discomfort expressed by the cry.

**Results:**

We show that low-pitched cries are more likely to be attributed to boys and high-pitched cries to girls, despite the absence of sex differences in pitch. Moreover, low-pitched boys are perceived as more masculine and high-pitched girls are perceived as more feminine. Finally, adult men rate relatively low-pitched cries as expressing more discomfort when presented as belonging to boys than to girls.

**Conclusion:**

Such biases in caregivers’ responses to babies’ cries may have implications on children’s immediate welfare and on the development of their gender identity.

**Electronic supplementary material:**

The online version of this article (doi:10.1186/s40359-016-0123-6) contains supplementary material, which is available to authorized users.

## Background

Stereotypical beliefs that sexes differ in their affective and cognitive abilities are pervasive and shape various aspects of human psychology and behaviour [3, 18, 38], with far-reaching consequences on the structure and functioning of societies [21, 23, 29, 33]. Gender stereotyping affects several dimensions of parental behaviour from the moment a child is born (e.g. parents dress baby boys and girls differently [43], encourage them to engage in different activities [8, 14], or expect them to perform differently when facing physical challenges [28]), thus contributing to the development of gender identity [24]. However, the extent to which gender stereotypes interfere with crucial dimensions of parental care, such as caregivers’ assessment of babies’ personalities and needs, has received little scrutiny [11]. In particular, although crying is a ubiquitous signal for human babies to communicate their distress and their needs [2, 42, 48, 49], whether inter-individual acoustic differences in cries affect caregivers’ gender attributions, and whether this affects their interpretation of the functional content of cries, has not yet been investigated. To address this gap, we conducted a series of acoustic analyses and listening experiments that investigate whether stereotypical expectations arising from sex differences in the voice of human adults influence how adult listeners perceive and assess babies’ cries.

According to the source-filter theory [5, 15, 45], the acoustic properties of the human voice mainly depend on the fundamental frequency (F0) imposed by the rate of vibration of the vocal folds inside the larynx and on the formant frequencies, imposed by the resonance characteristics of the supralaryngeal vocal tract [45]. The auditory perception of voice pitch depends mainly on mean F0, with a minor contribution of resonance characteristics [34]. Before puberty, the mean F0 of the voice does not differ between boys and girls [7, 10, 25, 44]. However as a result of a disproportionate increase in the length of the vocal folds in male adolescents during puberty, adult men speak on average with approximately 50 % lower F0s (and therefore lower-pitched voices) than women [22, 45]. Here, to test the hypothesis that adult listeners over-generalise this strong sex-dimorphism to their perception and interpretation of babies’ cries, we combined acoustic investigations and psychoacoustic experiments.

First, we analysed the acoustic structure of cries from 4-months old babies of both sexes to verify the absence of a sexual signature, and particularly the absence of sex differences in the F0 of the cries. Then, in order to assess whether adult listeners generalise sex differences in the F0 of adult voices to the cries of babies, we investigated whether F0 predicted sex attributions in natural cries. In order to isolate the effect of cry pitch we also exposed listeners to cries modified using the PSOLA re-synthesis method, which allows us to change the F0 of cries while keeping all other acoustic features unmodified [30]. We predicted that higher-pitched cries would be more likely to be attributed to girls, and lower-pitch cries to boys.

Second, we investigated whether individual differences in cry pitch also have an effect on adult listeners’ gendered attribution (masculinity in boys or femininity in girls). To do this we asked participants to rate the masculinity or femininity of babies’ cries, again using both natural and re-synthesised cries. We predicted that higher-pitched cries would be rated as more feminine and that lower-pitched cries would be rated as more masculine.

Third, we tested if the pitch of natural and re-synthesised cries predicted listeners’ perception of the degree of discomfort expressed by the baby. Based on previous evidence that intra-individual variation in cry pitch is a positive correlate of pain intensity [4], we predicted that discomfort ratings may be affected by inter-individual differences in cry pitch, with natural cries from higher-pitched babies rated as expressing more discomfort. We also hypothesised that sex-stereotypical biases may lead to differential expectation regarding boys’ or girls’ baseline pitches, that would in turn affect the level of disconfort perceived to be expressed by their cries. More specifically, we predicted that if listeners expect boys’ cries to be lower pitched than girls’, then, for a given pitch, discomfort should be perceived as higher when listeners are told that the cry originates from a boy than when they are told that it originates from a girl.

## Methods

### Recordings

We recorded spontaneous cries from 15 boys and 13 girls (we aimed to record between 12 and 15 children of each sex within the study period) of on average 4 months of age (M = 116 ± 21 days), while they were given their bath by their parents at home. Recordings were performed with a microphone (Sennheiser MD42) positioned approximately 30 cm from the baby’s face and connected to a Marantz PMD690/W1B recorder. To limit pseudo-replication, we recorded each baby during three independent sessions.

### Sound analyses

We isolated two sequences of crying from each recording session, resulting in a total of six crying sequences for each baby (mean sequence duration = 7.8 ± 1.1 s), and extracted a set of 15 temporal and spectral variables from each sequence. To describe the acoustic structure of cries, we used a dedicated batch-processing script in PRAAT [6], which contained four distinct procedures. These procedures have been applied successfully to the characterisation of acoustic variation in previous studies of babies’ cries [20].

The first procedure of the script characterized the fundamental frequency (F0 or pitch) and the intonation (F0 contour variation) of the cries. The F0 contour was extracted using the To Pitch (cc), command. The experimenter systematically inspected the extracted Pitch contour and verified it using a narrow band spectrogram displaying the first 2000 Hz of the signal. Spurious octave jumps were manually corrected by selecting the appropriate F0 candidate values in the edited pitch object. In the relatively rare segments including double vibration (where a weak subharmonic equal to half the fundamental frequency is present), the F0 was systematically preferred over the subharmonic. Each extracted F0 contour (pitch object) was saved as a text file for future reference. These numerical representations were used to derive the following parameters: *%voiced* (percentage of the signal that is characterized by a detectable pitch), *mean F0*, *max F0*, *min F0* (respectively the mean, maximum and minimum F0 calculated over the duration of the signal) and *F0CV* (coefficient of variation of F0 over the duration of the signal). In a second step, two distinct smoothing algorithms (*Smooth…* command in Praat) were performed on the pitch contour: the first allowed a relatively broad bandwidth (*Smooth…* command parameter = 25), to suppress very short-term frequency fluctuation while preserving minor intonation events (such as bleat-like frequency modulation), and the second only allowed a narrow bandwidth (*Smooth…* command parameter = 2), to only characterize strong F0 modulation (major intonation events). Inflection points were counted (as each change in the sign of the contour’s derivative) after each smoothing procedure, and divided by the total duration of the voiced segments in each recording, resulting in two distinct indexes of F0 variation (*inflex25* and *inflex2*).

The second procedure focused on the intensity contour and allowed the characterization of the variability of the cries’ intensity by calculating *intCV*, the coefficient of variation of the intensity contour estimated using the To intensity command in PRAAT.

A third procedure focused on the periodic quality of the signal and measured the harmonicity (*harm*, degree of acoustic periodicity, measured as the ratio of harmonics to noise in the signal and expressed in dB), an index of jitter (*jitter*, small fluctuation in periodicity measured as the average of ‘local’, ‘rap’ and ‘ppq5’ measures in PRAAT) and an index of shimmer (*shimmer*, small variation in amplitude between consecutive periods, measured as the average of ‘local’, ‘apq5’ and ‘apq11’ parameters in PRAAT).

The final procedure characterized the spectral envelope of the cry by applying a cepstral smoothing procedure (bandwidth: 900 Hz) to each crying sequence, followed by the extraction of the first four spectral prominences (*fsp*1, *fsp*2, *fsp*3, *fsp*4) of the resulting smoothed spectrum. Because babies’ cries can be strongly nasalized [41], and can contain biphonation phenomena [42] that can create resonance-independent broadband components, the measured spectral peaks cannot be safely considered as accurate measure of formant frequencies and are therefore termed spectral prominences. However, the observed values 1.2, 3.1, 5.7 and 8.6 kHz are consistent with the newborn/infant vocal tract length (~7.5 cm between 2 and 6 months; [46]) predicting vocal tract resonances at about 1.1, 3.3, 5.5 and 7.7 kHz).

### Statistical analysis of the acoustic structure

To investigate acoustic differences between boys’ and girls’ cries (study 1), we first performed a Principal Component Analysis to collapse the 15 acoustic parameters into two single composite scores (principal components PC1 and PC2). We then used two linear mixed effect models with PC1 and PC2 as dependent measures (fixed effect: “sex”; random effect: “baby identity”). *P* values were obtained from a likelihood-ratio test comparing the fit of full models with a null model lacking sex effect. We also compared each 15 acoustic parameters between sexes using a mixed model analysis with “sex” as fixed effect, “baby identity” as random effect, “age” and “weight” as covariates. Finally, we used a cross-validated and permuted Discriminant Function Analysis (pDFA) to assess the possibility of discrimination between both sexes. A training data set (2/3 of the cries from each individual) was used to generate linear discriminant functions on the basis of the 15 acoustic features describing the cries. The remaining 1/3 of the cries were used as a cross-validation set to measure the percentage of correctly classified cries. The mean effect size was calculated from 100 random iterations. To obtain the statistical significance of the effect size, we compared the percent correct obtained in the analysis to the distribution of percent correct values obtained by randomly assigning the sex to each baby. This distribution was obtained from 1000 randomly created data sets where the sex identity of each individual is permuted (permuted DFA) [26, 31]. All data were analyzed using R [39].

### Sound re-synthesis

**Fig. 1:**
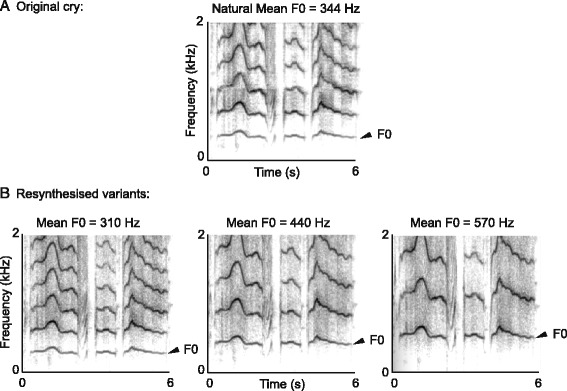
Illustration of PSOLA resynthesis applied to a baby’s cry. **a** Spectrogram of a natural cry with a mean fundamental frequency (F0) of 344 Hz. **b** spectrograms of resynthesised variants of this cry, with mean F0s set to 310 Hz, 440 Hz and 570Hz. While the F0 of the cry is scaled up or down, all other parameters in the call (duration, resonances, etc.) remain unchanged

One randomly selected cry from each of 24 babies (13 boys and 11 girls whose recordings were already available at the time of the playback experiment) was re-synthesised using the PSOLA algorithm (“Change Gender” command in PRAAT)[30]. PSOLA re-synthesis enables the independent rescaling of the Fundamental Frequency (F0, affecting the perceived pitch) while leaving all other parameters of the signal unchanged. PSOLA is a well established method for independently manipulating acoustic features in animal vocalisations (e.g. [40]) as well as human speech signals (e.g. [9, 16]). From each natural cry, we created a set of stimuli varying in their mean F0 only. We chose mean F0 values of 310 Hz, 375 Hz, 440 Hz, 505 Hz and 570 Hz, to fit to the mean cry pitch ± *n* SD (with *n* = 0, 1 and 2) as measured in our sample (Fig. 1).

### Playback experiments

The experiment aimed at testing the effect of cry pitch on sex attribution to natural cries by adult listeners (study 2) was performed using a Marantz PMD690/W1B recorder and Sennheiser HD 25-1 headphones. All participants were parents of 3-month-old babies (25 fathers and 27 mothers - we aimed at recording between 25 and 30 parents of each sex depending on recruiting availability). These participants were the parents of the babies whose recordings were used in the sound analyses and subsequent listening experiments. Each adult rated two successive experimental sets of cries, with 5 minutes separating the two sets. Each set included 12 cries: three different cries from each of four babies unfamiliar to the parent (two boys and two girls). The order of presentation of the cries was randomized and the adult listeners were unaware of the number of babies and of the sex ratio in the set of cries. Listeners were given the option to answer that they could not guess the sex of the baby. The playback test was conducted as a double-blind experiment.

The remaining psycho-acoustic experiments were performed in quiet rooms at the University Jean Monnet/Saint-Etienne or at the University of Sussex, from Dell (desktop) or Apple (laptop) computers using the Experiment Multiple Forced Choice tool in PRAAT. Stimuli were played via Sennheiser HD 201 Closed Back Headphones or Dynamode DH-660 headsets. Stimuli presentation was randomized and participants were invited to pause after every 12 ratings. First, participants entered each rating by clicking on the chosen button on the screen, then they could either confirm their choice (“OK” button), replay the sound (replay button) or change their rating (“oops” button).

To test if the pitch of the cry affected listeners’ sex attributions (study 3), we played back re-synthesised cry variants (120 stimuli) to 32 adult listeners (21 women and 11 men; 18 French parents followed by 14 undergraduate students in Psychology at the University of Sussex, attending a final year module; recruitment was terminated when the participation of the 14 undergraduate students brought the sample above our target of 30 participants). Participants were asked to identify the sex of the baby from listening to one of its cries.

All the subsequent experiments (studies 4 and 5) involved second year undergraduate students in Psychology at the University of Sussex following the Cognitive Psychology module. Participants only performed one of the four experiments (one type of rating), and were attributed to a given experiment by splitting the full sample of into four groups of approximately equal size, based on the initial of their name (listed in alphabetical order). Groups of participants were tested simultaneously during several practical sessions. The minimum sample of 30 participants for each experiment was reached for all experiments. All tested participants who terminated the experiment and provided an output data file were included in the analysis.

To test the hypothesis that cry pitch affects perceived gender attributes (masculinity in male babies and femininity in female babies, study 4), we used our set of natural cries and associated re-synthesised pitch variants in listening experiments where adult participants were asked to rate gender attributes of babies from listening to their cries. Thirty listeners (25 women and 5 men) were told that the cries were from 4 month-old baby boys and asked to rate their masculinity (on a Likert scale of 1 to 7: 1 = extremely low, 4 = average, 7 = extremely high). The question was: “Please rate the masculinity of this baby boy on a scale of 1 to 7: 1 = extremely feminine, 4 = neither feminine nor masculine, 7 = extremely masculine”. Thirty-eight different listeners (26 women and 12 men) were told that these cries originated from 4 month-old baby girls and asked to rate their femininity (also on a scale of 1 to 7). The question was: “Please rate the femininity of this baby girl on a scale of 1 to 7: 1 = extremely masculine, 4 = neither masculine nor feminine, 7 = extremely feminine”. Each adult rated a total of 24 natural cries, from 13 boys and 11 girls, and 120 re-synthesised cries, corresponding to the 5 pitch variants for the 24 exemplars (the presentation of natural and re-synthesised cries was randomized throughout).

To test the effect of cry pitch and declared baby sex on the perception of discomfort (study 5), different adult listeners were asked to rate the level of discomfort expressed by each cry, here too using a seven-point Likert scale. Two groups of participants were asked to rate our sets of 24 natural and 120 re-synthesised cries: one set of participants (30 women and 6 men) was told that the cries originated from boys, and the other (30 women and 11 men) that they originated from girls.

### Statistical analysis of the results of playback experiments

The effect of natural and artificial variation in acoustic parameters on listener’s ratings were tested using Linear Mixed Models (for continuous outcome variables: *femininity*, *masculinity* and *discomfort*) and Generalized Linear Mixed Models (with logistic regression link for the binary variable *sex*) in SPSS 21 for MAC. Reported statistics correspond to fully factorial models. Model structures are detailed in the footnotes of the Supplementary Tables. The sizes of main effects (fixed mean F0) or correlations (naturally varying meanF0) were estimated using R coefficients derived from simple linear regressions between the main meanF0 and the ratings averaged by exemplar and/or listener (*sex*, *femininity*, *masculinity* and *discomfort*).

## Results

### Study 1: Comparison of cry acoustics between sexes

**Fig. 2:**
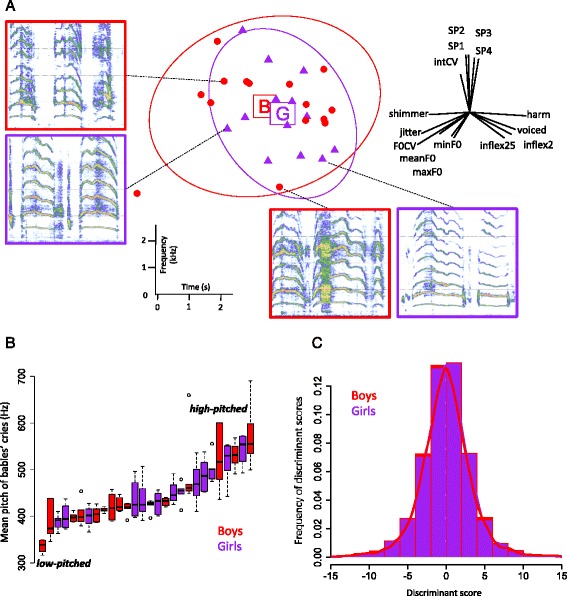
Absence of reliable sexual signature in human babies’ cries. **a** Principal component analysis (PCA). Each disk or triangle represents the centroid of one baby’s cries (red disks = boys; purple triangles = girls). The radar plot on the top left represents the loadings of the acoustic variables in the PCA circle of correlations. Spectrograms on the sides illustrate the similarity between girls’ and boys’ cries (Additional file 1). **b** The distribution of mean pitch (mean F0) does not significantly differ between the sexes (see Table 1). Boxplots display the distribution of cry pitch for each recorded baby, showing that both boys’ and girls’ cries are distributed widely on the pitch scale. **c** Frequency distribution of discriminant scores (Permuted Discriminant Function Analysis; mean discrimination success rate for the validating data set = 53.3 %, chance level = 50 %). *n*_*1*_ = 15 boys, *n*_*2*_ = 13 girls; boys in red, girls in purple (**a**-**c**)

The principal component analysis performed on the 15 acoustic variables characterizing the acoustic structure of babies’ cries highlighted a lack of differences between the cries of both sexes (Fig. 2a; respective Eigenvalues for PC1 and PC2 = 3.65 and 2.87; GLM on PC1: *χ*^2^ = 1.03, df = 1, *p* = 0.309; on PC2: *χ*^2^ = 0.233, df = 1, *p* = 0.629; *N* = 15 boys and 13 girls; 6 cries per baby; Additional file 1).

Univariate comparisons confirmed the absence of significant difference between the sexes for all the measured variables (all *p* > 0.05, Table 1). Specifically, pitch descriptors (min, mean and max F0) did not significantly differ between sexes (Table 1), with baby boys’ and girls’ ranges overlapping (Fig. 2b). Accordingly, the Discriminant Function Analysis testing for sex identification only resulted in a 53.3 % correct classification rate, which was not significantly different from chance (permuted DFA, *p* = 0.75; Fig. 2c).

**Table 1:** Comparison of acoustic variables between sexes

Variable	Boys’ cries (*mean* ± *se*)	Girls’ cries (mean ± *se*)	*F*	*p*
%voiced	61.5 ± 2.3	65.6 ± 2.4	1.55	0.224
min F0	268 ± 11	287 ± 11	1.46	0.238
mean F0	443 ± 16	454 ± 17	0.26	0.614
max F0	663 ± 36	645 ± 38	0.11	0.747
F0CV	0.09 ± 0.02	0.08 ± 0.02	0.29	0.596
inflex25	5.7 ± 0.4	6.1 ± 0.5	0.43	0.517
inflex2	0.83 ± 0.08	1.02 ± 0.09	2.46	0.129
harm	15.6 ± 0.7	16.3 ± 0.8	0.39	0.537
jitter	0.004 ± 0.001	0.003 ± 0.001	0.06	0.802
shimmer	0.032 ± 0.007	0.028 ± 0.008	0.13	0.721
intCV	1.66 ± 0.05	1.59 ± 0.06	0.70	0.411
fsp1	1286 ± 69	1249 ± 75	0.13	0.722
fsp2	3123 ± 147	3108 ± 158	0.01	0.946
fsp3	5697 ± 258	5853 ± 278	0.17	0.683
fsp4	8377 ± 363	8688 ± 390	0.34	0.565

Univariate Linear Mixed Models testing the effect of the sex of the baby on all 15 acoustic variables. The model included baby identity as a subject variable, and baby’s age and baby’s weight as random covariates (*n*_*1*_ = 15 boys, *n*_*2*_ = 13 girls). Degrees of freedom: 1 (numerator), 26 (denominator)

### Studies 2 & 3: Effect of cry pitch on sex attribution by adult listeners

Adult listeners asked to identify the sex of an unfamiliar baby from listening to one of its cries attributed a sex in 97.5 % of the trials (*n* = 1248). While the actual sex of the baby was a significant predictor of the attributed sex, neither the baby’s weight, the participant’s sex, nor the interaction term between the participant’s sex and the baby’s sex were significant predictors of the attributed sex (Table 2). The rate of correct identification was however only marginally higher than the chance level of 50 % (63.7 % for boys’ cries and 58.4 % for girls’ cries).

**Table 2:** Effect of baby’s sex on adult listeners’ sex attribution

Source	df	F	*P*
Intercept	4, 1190	10.44	<0.0005
Baby’s sex	1, 1190	33.26	<0.0005
Baby’s weight	1, 1190	0.03	0.875
Participant’s sex	1, 1190	0.17	0.679
Participant’s sex * Baby’s sex	1, 1190	0.03	0.868

Generalized Linear Mixed Model (GLMM), with binary logistic regression link testing the effects of baby’s actual sex, baby’s body weight, participant sex, and baby’s sex * participant’s sex on participants’ attribution of sex to natural babies’ cries. Participant identity is included as subject variable and recording session (baby’s identity) as a nested random factor term. Baby’s sex is a highly significant, but very poor predictor of attributed sex

**Fig. 3:**
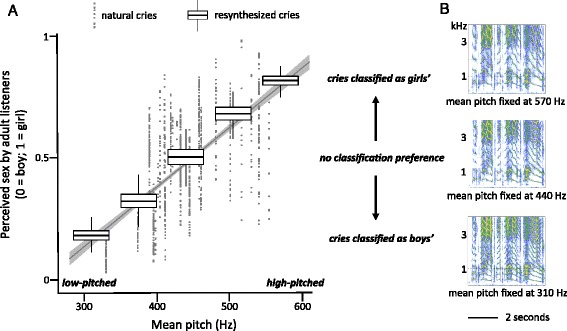
Pitch-dependent attribution of baby’s sex by adult listeners. **a** Dots represent the sex attributed by adults participants (25 men and 27 women) listening to natural cries of unfamiliar babies (14 boys and 11 girls) with different pitch profiles (x-axis: baby’s mean F0, averaged over 6 cries; y-axis: baby’s estimated sex attribution; fitted line: marginal mean ± SE). Boxplots represent the average sex attribution given by adult listeners (11 men and 21 women) to each of five mean pitch re-synthesis variants (x-axis: mean F0 variants = 310, 375, 440, 505 and 570 Hz; y-axis: pitch variant’s estimated marginal mean sex attribution). **b** Examples of re-synthesised variants from the same cry exemplar. Only the pitch is affected by the PSOLA algorithm used for re-synthesis (Additional file 2)

In playbacks involving natural cries, sex attributions by adult listeners varied significantly between babies (Table 3), with some babies consistently identified as male and others consistently identified as female (Fig. 3; Additional file 2).

**Table 3:** Inter-individual differences in attributed sex between babies

Source	*df*_*1*_ *, df*_*2*_	*F*	*p*
Intercept	24, 1170	5.95	<0.0005
Baby’s identity	24, 1170	5.95	<0.0005

Generalized Linear Mixed Model (GLMM) with binary logistic regression link testing the effect of baby’s identity on participants’ attribution of sex to natural babies’ cries. Participant identity is included as subject variable and recording session as a random factor term

In trials involving natural cries, MeanF0 (and to a lesser extent shimmer) was a significant predictor of attributed sex (Table 4a). Higher-pitched cries were significantly more likely to be identified as belonging to girls, and lower-pitched cries were significantly more likely to be attributed to boys (Fig. 3). This effect was intermediate (*R* = 0.43, as estimated from simple linear regression of the average sex rating (coded as male = 1 and female = 2) by exemplar over meanF0).

**Table 4:** Effect of acoustic variables on sex identification for natural and re-synthesised cries

Source	*df*_*1*_ *, df*_*2*_	*F*	*p*
a. Rating of natural cries			
Intercept	15, 1179	6.18	<0.0005
%voiced	1, 1179	1.27	0.260
Pitch (=mean F0)	1, 1179	30.03	<0.0005
maxF0	1, 1179	0.99	0.320
minF0	1, 1179	0.04	0.838
Inflex25	1, 1179	3.27	0.071
Inflex2	1, 1179	2.57	0.109
F0CV	1, 1179	2.41	0.121
INTCV	1, 1179	0.51	0.476
harm	1, 1179	2.10	0.147
jitter	1, 1179	2.68	0.102
shimmer	1, 1179	4.67	0.031
fsp1	1, 1179	3.30	0.070
fsp2	1, 1179	1.50	0.221
fsp3	1, 1179	1.31	0.254
fsp4	1, 1179	0.40	0.526
b. Rating of re-synthesised cries			
Intercept	9, 3830	80.77	<0.0005
Participant sex	1, 3830	2.63	0.105
Pitch	4, 3830	164.69	<0.0005
Participant sex * Pitch	4, 3830	0.80	0.528

(a) Generalized Linear mixed model (GLMM) with binary logistic regression link testing the effect of all 15 acoustic variables on participants’ attribution of sex to natural babies’ cries. Participant identity is included as a subject variable, and recording session (baby’s identity (baby’s actual sex) as a nested random factor term. (b) GLMM with binary logistic regression link testing the effects (main and interactions) of Participant sex and Pitch (=mean F0) on participants’ attribution of sex to re-synthesised babies’ cries. Participant identity (tested population) is included as nested subject term, and cry exemplar (recording session (baby’s identity (baby’s actual sex) as a nested random factor term

In trials involving re-synthesised cries, fixed meanF0 had a significant effect on attributed sex (Table 4b): adults identified re-synthesised cries with a meanF0 fixed at 310 Hz as belonging to boys in 82.5 % of the trials, and cries with a mean pitch fixed at 570 Hz as belonging to girls’ in over 82.3 % of the trials (Fig. 3).

### Study 4: Effect of cry pitch on masculinity and femininity ratings by adult listeners

**Fig. 4:**
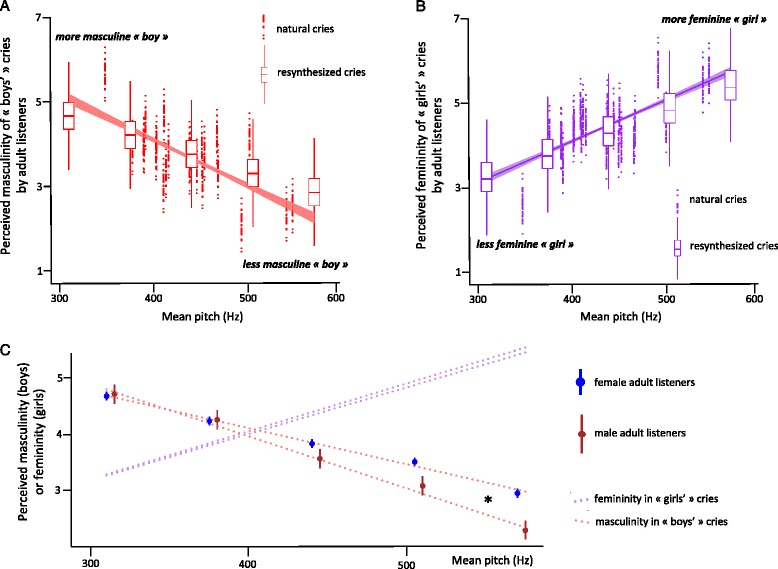
Cry pitch drives the baby’s perceived gender by adult listeners. **a** Red dots represent the perceived gender by adults (25 women, 5 men) listening to cries presented as originated from boys (x-axis: baby’s mean F0; y-axis: baby’s estimated gender attribution; solid line: linear fit of the estimated marginal means ± SE). Red boxplots represent the average perceived gender given by adults to each of five mean pitch re-synthesis variants presented as originated from boys (x-axis: mean F0 variant; y-axis: pitch variant’s gender attribution). **b** Purple dots represent the perceived gender by adults (26 women, 12 men) listening to cries presented as originated from girls (x-axis: baby’s mean F0; y-axis: baby’s estimated gender attribution; solid line: estimated marginal means ± SE). Purple boxplots represent the average perceived gender given by adults to each of five mean pitch re-synthesis variants presented as originated from girls (x-axis: mean F0 variant; y-axis: pitch variant’s gender attribution). **c** Interaction between adult listener sex and cry pitch on gender rating. Dots represent pitch variant’s estimated marginal mean ± SE (blue dots: adult women; brown dots: adult men; red dotted lines: linear fits of the estimated marginal means for cries presented as belonging to boys; purple dotted lines: linear fits of the estimated marginal means for cries presented as belonging to girls). * *p <* 0.05

In trials involving natural cries, cry pitch was a significant predictor of ratings of femininity and masculinity: natural MeanF0 was a significant negative predictor of perceived masculinity in boys (Table 5a and Fig. 4) and a significant positive predictor of femininity in girls (Table 5c and Fig. 4). Effect sizes associated with simple linear regressions assessing these correlations were large (*R* = 0.66 in both cases).

**Table 5:** LMMs testing the effect of experimental factors on ratings of cry gender

Source	*df*_*1*_ *, df*_*2*_	*F*	*p*
a. Masculinity rating of natural cries			
Intercept	1, 712.7	186.18	<0.0005
P. sex	1, 712.7	0.068	0.795
Pitch	1, 688	63.94	<0.0005
P. sex * Pitch	1, 688	0.31	0.577
b. Masculinity rating of re-synthesised cries			
Intercept	1, 28	2438.57	<0.0005
P. sex	1, 28	2.96	0.096
Pitch	4, 2872	182.02	<0.0005
P. sex * Pitch	4, 2872	6.18	<0.0005
c. Femininity rating of natural cries			
Intercept	1, 908	0.86	0.355
P. sex	1, 908	1.47	0.226
Pitch	1, 872.8	87.18	<0.0005
P. sex * Pitch	1, 872.8	1.68	0.196
d. Femininity rating of re-synthesised cries			
Intercept	1, 34.8	3882.55	<0.0005
P. sex	1, 34.8	0.18	0.676
Pitch	1, 3669	468.42	<0.0005
P. sex * Pitch	1, 3669	0.80	0.528

Linear mixed models (LMM) testing the (main and interaction) effects of participant sex (P. sex) and cry mean F0 (Pitch) on participants’ ratings of masculinity in cries presented as belonging to boys and femininity in cries presented as belonging to girls (both rated along a 7 point Likert scale). Natural cries: LMM includes participant identity as subject variable, and actual baby’s sex as a random factor. Re-synthesised cries: LMM includes participant identity as subject variable, and baby identity (actual baby’s sex) as nested random factors

In trials involving re-synthesised cries, fixed meanF0 also had significant effects on perceived masculinity or femininity: lower-pitched cries presented as belonging to boys were rated as more masculine (Table 5b and Fig. 4), and higher-pitched cries presented as belonging to girls were rated as more feminine (Table 5d and Fig. 4). There was a significant interaction between listener’s sex and re-synthesised pitch on masculinity ratings (but not on femininity ratings): men rated higher-pitched cries from boys as significantly less masculine than women did (Table 5b and Fig. 4).

### Study 5: Effect of cry pitch on discomfort attributions by adult listeners

**Fig. 5:**
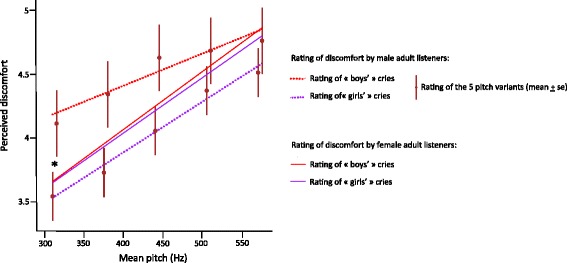
Influence of cry pitch and of baby’s declared sex on adult listeners’ assessment of discomfort levels. The figure shows the perceived discomfort (rated on a seven-point Likert scale) during listening of five pitch variants of re-synthesised cries (mean F0 = 310, 375, 440, 505 and 570 Hz, respectively). One set of participants (30 women and 6 men) was told that the cries originated from boys, and the other (30 women and 11 men) that they originated from girls. Higher-pitched cries are rated as expressing more discomfort than lower-pitched cries (effect of re-synthesised pitch variant on rating score: F(4,7376) = 188.8, *P* < 0.0005). At lower pitch, male listeners over-estimate the discomfort expressed by cries when presented as belonging to boys (red dots) compared to cries presented as belonging to girls (purple dots; F(4,7376) = 2.5, *P* = 0.04). For the purpose of clarity, only the fitted lines of the marginal means are shown for female listeners (red and purple solid lines). * *p <* 0.05

In trials involving natural cries, meanF0 was a significant predictor of discomfort ratings (Table 6a). Adult listeners rated higher-pitched cries as expressing more discomfort than lower-pitched cries (Table 6a and Fig. 5). This effect was intermediate (*R* = 0.35, as estimated from simple linear regression of discomfort rating over meanF0).

**Table 6:** LMMs testing the effect of experimental factors on ratings of cry discomfort

Source	*df*_*1*_ *, df*_*2*_	*F*	*p*
a. Rating of natural cries			
Intercept	1, 1806	38.42	<0.0005
Participant sex	1, 1806	2.14	0.144
Declared baby sex	1, 1806	0.05	0.816
Pitch	1, 1767	39.22	<0.0005
Participant sex * Declared baby sex	1, 1806	1.90	0.168
Participant sex * Pitch	1, 1767	2.21	0.138
Declared baby sex * Pitch	1, 1767	0.40	0.529
Participant sex * Declared baby sex * Pitch	1, 1767	2.49	0.115
b. Rating of re-synthesised cries			
Intercept	1, 73	2453.91	<0.0005
Participant sex	1, 73	0.08	0.784
Declared baby sex	1, 73	2.12	0.152
Pitch	4, 7376	188.79	<0.0005
Participant sex * Declared baby sex	1, 73	1.53	0.219
Participant sex * Pitch	4, 7376	4.86	0.001
Declared baby sex * Pitch	4, 7376	2.12	0.075
Participant sex * Declared baby sex * Pitch	4, 7376	2.51	0.040

Linear mixed models (LMM) testing main and interaction effects of participant sex, declared baby sex, cry Pitch (=mean F0) on participants’ ratings of discomfort (7 point Likert scale). (a) Natural cries: LMM includes participant identity as subject variable, and actual baby’s sex as a random factor. (b) Re-synthesised cries: LMM includes participant identity as subject variable, and baby identity (actual baby’s sex) as nested random factors

In trials involving re-synthesised cries, fixed meanF0 had significant effect on perceived discomfort, with higher pitched cries rated as expressing more discomfort in both sexes (Table 6b and Fig. 5). There was a significant three-way interaction between the sex of the listener and both the declared sex of the baby and the pitch of the cry: while female participants gave similar discomfort ratings to putative boys’ cries and putative girls’ cries, male participants rated putative boys’ cries as expressing more discomfort than putative girls’ cries, particularly for lower-pitched variants (Table 6b and Fig. 5). In order to verify that this result did not arise from the sex imbalance that characterises our sample of listeners, we re-ran this analysis with female listeners only and with male listeners only. The results confirm that no effect of declared baby sex is found in female listeners (F_(1, 1438)_ = 0.36, *P* = 0.55). They also confirm that men attribute higher levels of discomfort when they are told that the cry originates from a boy (F_(1, 406)_ = 18.63, *P* < 0.001), and that they do so particularly when the cry is low-pitched (F_(4, 1624)_ = 2.93, *P* = 0.02).

## Discussion

Together, our results indicate that despite the absence of measurable sex differences in the pitch of babies’ cries, adult listeners generalized the sex dimorphism that characterizes the voice of adult speakers to their perception of the cries, and that this not only influenced their attribution of sex and gender-related traits to crying babies, but also to some extent their assessment of the babies’ discomfort.

### Effect of cry pitch on listener’s sex attribution (studies 1, 2 & 3)

Our detailed acoustic analyses showed that the acoustic structure of human babies’ cries does not convey reliable information on their sex. None of the measured variables (either related to the glottal source or to the vocal tract filter) differed between boys and girls, as expected from the absence of sex differences in the morphology and dimensions of the newborn and infant vocal apparatus [46]. In particular, the variables characterising the pitch of the cry did not vary between sexes, corroborating previous investigations of sex difference in infant cries’ pitch [19, 32]. While the results of the discriminant analysis based on linear combinations of these acoustic variables failed to identify the sex of the babies better than chance, adult listeners performed marginally higher than chance when asked to identify the sex of the cry. This suggests that listeners may rely on sex differences in the dynamic variation of acoustic parameters or on nonlinear combinations of these parameters that were not captured in our acoustic analyses to make their judgements. However, while recent acoustic investigations of infants’ cries found an effect of sex hormones on the fundamental frequency contour, they did not identify sex differences [47]. Furthermore, it is important to note that this discrimination was highly unreliable, as the rate of error in listeners’ sex recognition remained very high at approximately 40 % (in other words, the rate of correct sex attribution was only marginally better than the 50 % chance rate for both sexes).

Crucially, despite the absence of sex differences in cry pitch, this key acoustic dimension was a strong predictor of the sex attributed to the cry, with naturally higher pitched cries more likely to be attributed to girls, and naturally lower-pitched cries more likely to be attributed to boys. Moreover, the effect of the pitch on sex attribution was confirmed experimentally using re-synthesised cries: cries with artificially raised pitches were more likely to be characterised as belonging to a girl and vice versa, independently of any other acoustic parameter. This suggests that adult listeners generalize the sexual dimorphism characteristic of adult voices to the cries of 3-month-old babies. Such over-generalisations, which are common in human perception of nonverbal vocal cues (e.g. listeners typically expect lower pitched men to be taller, despite the absence of reliable relationship between F0 and size in adult men [37]), are likely to reflect stereotypical biases which may interfere with the decoding of more functional information in the signal.

### Effect of cry pitch on attributions of femininity and masculinity (study 4)

Inter-individual variation in cry pitch also predicted listeners’ characterization of unfamiliar babies’ gender attributes from their cries, with low-pitched boys’ cries perceived as belonging to more masculine boys and high-pitched girls’ cries perceived as belonging to more feminine girls; and here too, the effect of meanF0 was confirmed experimentally using re-synthesised cries. Voice pitch has previously been found to predict masculinity and femininity in adults [36, 37], but also, and more surprisingly, in pre-pubertal children’s voices, again despite the absence of between-sex differences in F0 at this age [9]. The fact that men rated higher-pitched re-synthesised cries from boys as significantly less masculine than women did could result from differential exposure resulting in female listeners being better experts [20], or to the fact that male listeners apply more sex stereotypical biases to boys’ vocalizations, in line with indications that men may succumb to sex stereotypes more than women [13]. While these results are statistically significant, it is important to note that the sex imbalance in our experimental groups invites caution, and that these results should be confirmed by further investigations using a more balanced set of listeners.

Together these investigations provide the first experimental demonstration that cry pitch strongly influences the perception of gender attributes in unfamiliar babies’ cries. They suggest that caregivers may attribute gender profiles to babies on the basis of the quality of their voice, even at an age when masculinity and femininity may have relatively little functional relevance (though see [1]). Further studies should however investigate the medium and long-term stability of inter-individual variation in F0, and how this relates to inter-individual differences in gonadal hormone profiles, as well as personality traits, including gender attributes. Indeed we cannot exclude that these (sex-independent) inter-individual differences in cry pitch may reflect differential exposure to gonadal hormones during prenatal or early postnatal development [47], and therefore function as cues to vocal femininity and masculinity in infants as well as throughout the lifespan of the individual. Given the importance of social environment and experience in the development of child personality, the effect of sex stereotypes on adult listeners’ perception of babies’ cries reported here may directly affect the emergence of children’s gender identity, with caregivers providing differential feedback to babies [17, 27]. For instance, the fact that high-pitched boys are perceived as less masculine raises the possibility that adults may adjust their caregiving behaviour in accordance with other gender stereotypes, e.g. by engaging them in fewer “boyish” activities (Endendijk et al. 2014 [14]), or expecting them to perform poorer during physical activities [28]. Future work should now examine caregivers’ attitudes towards babies characterised by different cry pitch profiles to investigate the possible role of this parameter in the development of gender identity.

### Effect of cry pitch and declared baby sex on attributions of discomfort to unfamiliar babies (study 5)

We found that adult listeners rated natural cries from higher-pitched babies as expressing more discomfort than cries from lower pitched babies. The specific effect of pitch on perceived discomfort was demonstrated by the ratings attributed to re-synthesised cries. While intra-individual variation in cry pitch has been identified as a positive correlate of pain intensity (e.g. [4]), as well as a key factor driving parents’ assessment of the level of discomfort expressed by cries [12, 49], to our knowledge the effect of inter-individual variation in cry pitch [20] on discomfort assessment had never been investigated. The fact that higher-pitched cries were rated as expressing more discomfort raises the possibility that unfamiliar caregivers may overestimate the discomfort of babies presenting a higher-pitched voice (and underestimate discomfort in lower-pitched babies). When asked to rate levels of discomfort in natural cries of unfamiliar babies, listeners were not affected by the declared sex of the baby. However, in the ratings of re-synthesised cries, we found a significant interaction between the sex of the listener, the declared sex of the baby and the pitch of the cry: while female participants gave similar discomfort ratings to putative boys’ and putative girls’ cries, for lower-pitched variants male listeners rated putative boys’ cries as expressing more discomfort than putative girls’ cries (Fig. 5; Extended Data Table 6). This interaction effect may indicate that sex-stereotypical expectations that male babies should be lower-pitched than female babies lead male listeners to overestimate discomfort in unfamiliar boys’ cries. One would expect that parents who are familiar with their baby build a referential scale that allows them to accurately assess her/his cries [20]. However, in contexts where babies are placed with unfamiliar persons, such biases may have a tangible impact on care provision, which could be moderated by raising awareness of the potential impacts of sex stereotypes on cry perception, as well as by favouring more stable interactions between caregivers and individual babies [35].

## Conclusion

Our results illustrate how sex-stereotypes arising from the generalisation of sex differences in voice pitch that characterise adults’ voices, but not babies’ cries, drive elusive perceptual biases that may lead adults to attribute gender traits and thereby bootstrap the construction of individual gender identity. Future studies should examine the effect of interindividual difference in endorsement of gender stereotypes on these biases, as well as the extent to which these stereotypes interfere with crucial aspects of adults’ immediate assessment of the babies’ condition by unfamiliar male listeners, and whether this translates into differential treatment. We suggest that these potentially detrimental biases could be brought to the attention of new parents, as well as early childhood professionals.

### Declarations section

#### Ethics

Studies conducted in France were performed under the authorization n° 42-218-0901SV09 (ENES Lab, DDSVL) and approved by the local CNIL committee. Informed consent was obtained from all adult subjects and from the babies’ parents. Studies conducted in the United Kingdom were performed under authorization ER/REBY/1 [DR0213] from the Sciences and Technology Cross-Schools Research Ethics Committee of the University of Sussex.

### Consent to publish

Not applicable.

### Availability of data and materials

Complete data from this study can be obtained by contacting the corresponding author.

## Additional files

Additional file 1: Recordings of cries from four different babies (two boys and two girls). These sounds correspond to the spectrograms displayed in Fig. 1. (WAV 2637 kb) Additional file 2: Recordings of re-synthesised cry variants from the same baby (mean pitch fixed at 310, 440 and 570 Hz). These sounds correspond to the spectrograms displayed in Fig. 2. (WAV 2085 kb)
